# Influence of boarding secondary school environment on HIV positive students in South Western Uganda

**DOI:** 10.1186/s12889-021-10380-0

**Published:** 2021-02-09

**Authors:** Raymond Bernard Kihumuro, David Jolly Muganzi, Elton George Wandira, Racheal Alinaiswe, Jovitah Joselyne Nanyunja, Ruth Kugumisiriza, Paul E. Alele, Vincent Mubangizi

**Affiliations:** Mbarara University of Science and Technology, P.O. Box 1410, Mbarara, Uganda

**Keywords:** HIV, Boarding, School, Adolescents, Adherence, Stigma, Uganda

## Abstract

**Background:**

The number of human immunodeficiency virus (HIV) positive adolescents in secondary school has increased over the years. Little is known on how the students cope to the pressures and demands of their academic and health lives in the boarding secondary schools. This study explored the factors surrounding their anti-retroviral therapy adherence as well as their experiences.

**Methods:**

We did a qualitative study that employed in-depth interviews amongst purposively selected 19 HIV positive adolescent students in boarding secondary school and seven key informants. Key informants were members of boarding secondary school staff directly taking care of the adolescents living with human immune virus and had spent at least two academic terms in that school.

The study participants were recruited from four health facilities in Bushenyi district, southwestern Uganda, and key informants from five boarding secondary schools in Bushenyi. These were engaged in in-depth interviews using an interview guide. Data was transcribed, coded and the content analyzed thematically.

**Results:**

Adolescents living with human immunodeficiency virus in boarding secondary school face challenges similar to adolescents outside boarding school settings. However, some challenges are unique to them. Students faced numerous barriers which made it difficult to adhere to their medication. Stigmatization in its different forms was also a major challenge amongst students. Willingness disclosure of serostatus was beneficial to the students since it guaranteed support while at school; facilitating adherence and better living. However, students were uneasy to disclose their status. Some students adopted negative coping mechanisms such as telling lies, escaping from school, and class to access medication.

**Conclusions:**

Adolescents in boarding secondary schools face similar challenges as compared to their counterparts with some being unique to them. Few school mechanisms help these students to cope while at school. Limited disclosure has proven useful but some adolescents have opted not to disclose their status and hence used negative coping mechanisms. These challenges need to be addressed and a safe environment to encourage limited disclosure should be made.

## Background

Globally, adolescents make 4% of the HIV (human immunodeficiency virus) infection burden, with 89% of the infected adolescents found in sub-Saharan Africa [1]. Adolescents account for 55% of people living with HIV in Uganda [2]. Advent of anti-retroviral therapy (ART) has enabled HIV infected people to live a long life [3]. In Uganda, 55% of the population are below 19 years and majority of the secondary school going population are between 13 to 19 years [2].

Studies have shown that ALWH face challenges such as serostatus disclosure, stigma and ART adherence [3–7]. Adolescents get support from their families to overcome some of the challenges. Students in boarding school spend most of their time at school away from their families. School just like home is a salient socio-ecological context for adolescents’ well-being and development [6]. School is supposed to play a key role in ‘substituting for families [8]. Studies have shown that HIV positive school going children are negatively impacted by schools because they expose them to bullying and stigma [9].. Also, majority of schools lack an organized mechanism to care for HIV infected students [6, 10]. Furthermore, limited privacy and status disclosure may influence stigmatization and disrupt ART adherence [5, 8, 11, 12]. The situation could even be more complicated in a boarding school environment where there is limited privacy and there is no family support.

There are few studies done in sub-Saharan Africa on the influence of boarding secondary school environment on the experiences of ALWH, facilitators and barriers to ART adherence. We investigated this from the perspective of the HIV positive adolescent students and school staff who are directly responsible for caring for ALWH while in secondary boarding schools in Uganda. Our study findings provide ground for strengthening supportive school environments, with implications for policies and support programs that are aimed at mitigating the burden of the pandemic on the infected students thus meeting their educational, social and health needs as well as increasing ART adherence while in boarding school.

## Methods

### Study design

This is a qualitative cross-sectional study employing in-depth interviews which was conducted in July and August 2020.

### Study setting

The study was done in Bushenyi district, in south western Uganda. This region has a high HIV prevalence of 7.9% compared to the Uganda’s national prevalence of 6% [13]. The headquarters of the Bushenyi district is 321 km from Kampala city via Mbarara town. It has a total population of 234,440 with 116,410 males. The adolescents (10–19 year olds) comprise approximately 30% of the population [2]. In this region, the local economy is largely dominated by subsistence agriculture, animal husbandry, and petty trading. Food and water insecurity are common [14, 15].

The study was done in four health facilities namely Kyabugimbi Health centre IV, Kyamuhunga health centre III, St Daniel Comboni hospital and Ishaka Adventist hospital. The health centres were public while the hospitals were private not for profit ones. Each of the health facility run a special clinic for HIV-infected adolescents. Bushenyi district does not have a public hospital. Key informants were recruited from five boarding secondary schools.

We purposively selected schools basing on the type and setting of the school from which we recruited seven key informants. Bishop Ogez high school is a government-owned, rural-based mixed day and boarding school. Kyeizooba Girls’ school is a government-owned, rural-based girl only fully boarding school. St Mary’s Vocational school, Kyamuhanga is a privately owned, rural-based mixed day and boarding school. St Kagwa high school is a government-owned, town-based mixed day and boarding school. Ishaka Adventist college is a government-owned, town-based mixed fully boarding.

### Study population

Our study population was HIV positive adolescents who were in boarding secondary schools and had spent at least two academic terms (about 6 months) in the boarding secondary school.

The key informants were members of the school staff that were responsible for looking after ALWH and had been in the same school for about 6 months.

### Sampling and data collection

We purposively selected the health facilities and schools from which we recruited participants. We selected two public and two private health facilities basing on prior knowledge of the HIV adolescent clinics. We decided to recruit participants from health facilities instead of boarding schools to avoid inadvertently disclosing participants’ HIV status to classmates and school authorities in case students did not wish to have their status disclosed. This was also in accordance with the advice given to us by the Mbarara University Research Ethics committee.

After getting the administrative permission from the Bushenyi district administration, we explained the study with emphasis on inclusion and exclusion criteria to the in-charge of ART adolescent clinic. The inclusion criteria were HIV positive students aged 12–19 in boarding secondary schools in Bushenyi district and must have been in the same school for at least two terms. On the other hand, the exclusion criteria were HIV positive students who were acutely ill or hospitalized at the time of the study and HIV positive students who did not know their HIV status. The in-charge was then asked to identify and invite for us the potential participants. Using the ART clinic register, the in-charge identified the potential participants whom she then contacted using a phone and invited to them come to the clinic for a possible interview. We recruited six participants each from St Daniel Comboni hospital and Ishaka Adventist hospital, five and two participants from Kyabugimbi Health centre IV and Kyamuhunga health centre III respectively..

We purposively selected schools basing on the type and setting of the school from which we purposively recruited seven key informants. We introduced the study to the head of the respective schools and asked them to identify for us the key informants. We recruited one key informant from the following schools; Bishop Ogez high school, Kyeizooba Girls’ school and St Kagwa high school. We recruited two key informants each from St Mary’s Vocational school, Kyamuhanga. and Ishaka Adventist college.

### Interviewers

The interviewers were Health Professional Education Partnership Initiative -Transforming Ugandan Institutions Training Against HIV/AIDS (HEPI-TUITAH) micro-research grantees. They were students at Mbarara University in the faculty of Medicine. The interviewers had not established any relationship with the interviewees prior to the interviews.

### Data collection tool

We specifically developed interview guides to collect data through in-depth face to face interviews using open-ended questions, followed by probes in line with the study objectives. Both the tool for the participants and key informants were designed to collect data on the; demographic data of the participants, influence of boarding secondary school on the lives ALWH; facilitators and barriers of highly active ant-retroviral therapy adherence as well as experiences of ALWH. The questions in the interview guide were based on literature.

### Data collection procedure

We conducted in-depth interviews. The participants were identified from the study site with the help of the health facility staff and head teachers. The research team explained the study to potential participants and invited them to participate. All those who were invited to participate agreed. Participants gave written consent. For participants who came alone and were below 18 years, we considered them empowered minors (defined as those who were responsible for their own HIV care) and thus able to give consent to the study. Participants below 18 years who came with their parents, we considered them non-emancipated and thus sought written consent from their parents and then accent from adolescent.

Participants were then interviewed individually face to face using an interview guide in a private room at the study site between July and August 2020. Each interview was conducted until there was no new information generated. Approximately each interview lasted 30–40 min. The interviews were conducted in either English or Runyankole (local language), depending on the participant’s preference. Interviews were audio recorded and backed by field notes with participant’s consent.

Data collection was done by three researchers (RBK, DJM and EGW) and one research assistant. Two research teams were used to collect data concurrently. One member interviewed while the other was recording the interview with an audio recorder and taking notes. The team members alternated roles with subsequent interviews.

Interviews were conducted until no new information emerged from the process (saturation). Saturation was considered by consensus within the team as the point when no new information or emerging themes were obtained from each category of study participants with subsequent interviews. Saturation was reached at 19 for adolescent students and at seven for key informants.

### Data management and analysis

The interviews were recorded on a digital voice recorder and notes taken during the interviews. At the end of each data collection day, the interviews were listened to and modified the interview guide to suit the study better. At the end of the data collection, recorded interviews in English were transcribed verbatim into Microsoft word documents. Interviews in the local language were translated and transcribed into English. The transcripts were cleaned and anonymized. Processed data were then entered into Atlas. ti version 7 (qualitative analysis software for Windows) for coding and analysis. Data was read repeatedly and extensively from which a coding frame was developed and agreed on by two researchers (RBK and VM). Data were coded and analyzed thematically. The coding of the data was done by two researchers. Content analysis of the data was done using the deductive and inductive approach.

### Ethical approval

Ethical approval for this study was obtained from Mbarara University of Science and Technology Research Ethics Committee (Ref: 07/01–20). The study was cleared by the Uganda National Council for Science and Technology in line with national guidelines. Participants aged 18 years and above provided written consent. For participants who came alone and were below 18 years, we considered them emancipated minors and thus able to give written consent to the study. Participants below 18 years who came with their parents, we considered them non-emancipated and thus sought written consent from their parents and then the accent from adolescents.

## Results

Five broad themes emerged from the study of ALWH in boarding secondary schools namely; setback of HIV serostatus disclosure (discrimination and negative treatment); negative coping (unwillingness to disclose serostatus and its implications); stigma and its effects; facilitators of ART adherence, and barriers of ART adherence. Issues surrounding HIV status disclosure were intertwined in all themes. We interviewed 19 student participants aged 14 to 19 years. We recruited two to five participants from each class of senior two to six. Of the participants recruited 14 were females. We also interviewed seven key informants who were either school nurses, teachers, or matrons. All key informants were females and had spent between five to 13 years in their current school.

### Setbacks of HIV serostatus disclosure: discrimination and negative treatment

There were negative experiences reported by ALWH. These included being discriminated against, neglected, bullied, teased as a result of being HIV positive, gossiped about, insulted, assaulted, harassed, and being threatened on account of living with HIV/AIDS by either fellow students or staff.

*“ … because in the first school I used to keep my medicine along with me … there was a time where the students tried to open my case and when they opened my case, they saw medicine … they got them out … they never took them outside the dormitory but they hanged them there on my case. But then there is a friend of mine … He told me “something has happened in the dormitory because I hear students making shouting … when I went there I said “hey” … I saw my things hanged and I really was shocked … (Long pause).”* Participant 15, Male 17 years old, senior 2.

*“... Some teachers are very funny, they pretend to be* as *students. Also*, *they see it as something new to them though they have studied and done what … but they don’t do anything about us … You find them even talking about you instead of giving you advice … ”* Participant 18, Female 18 years old, senior 6.

Students who also engaged in leadership reported discrimination if their status was known. Some students reported being de-campaigned on the grounds of being HIV positive.

*“So of course, I could not feel okay when, while going de-campaigning me. (Yeah) No, I felt so bad. Even I called some* of *my friends; how shall we go about this thing? They said no, continue. Even there is when I wanted to say ah why should I; why should not write a letter of resignation saying that I should stop.”* Participant 01, Male 18 years, senior 4.

However, some students were uneasy with disclosing their status but had no way out.

*“When I wanted to come here (referring to health centre) to get my medicine so I decided that I should tell her (school nurse) the truth. I had asked for permission to come here so many times so she asked me, “Why do you like going home?” umm then I ended up telling her the truth.”* Participant 06, Female 16 years, senior 4.

*“There was a school nurse on the day when I reported, and as they were checking my suitcase at the gate, they found my drugs in my suitcase. They asked me what are the drugs for, and I told them.”* Participant 19, Female 19 years old, senior 6

### Negative coping: ALWH unwillingness to disclose serostatus and its implications

Some ALWH preferred to conceal their HIV status to avoid being stigmatized by their peers and staff members. They tried to avoid unintended disclosure of their HIV status by taking their pills in hiding and/ or even changed the package of the medicines. They also had to tell lies, escape, or give false reasons to be able to access their pills. Equally, they experienced the difficult to get permission to stay longer or access their dormitory to take their medications.

“*So, you find the child “dying” there in the dormitory struggling* with *how they are going to take the tabs. The dormitories are locked, they tell lies that matron I am in my menses I have come to pick my pad. The matron opens thinking that the person is picking a pad when she has come to take tablets, so that is the problem.”* Key informant 02, health teacher.

*“The director used to give me permission, sometimes I used to escape … … in the morning early from school when the day students are entering I would go out.”* Participant 13, Female 18 years old, senior 4.

“*When I am reporting, I get my skirt. It has the pocket (describes inner pockets of her skirt), then I keep my drugs in the small polythene so that they cannot shake and they make* a *sound. Then I keep down there in my case then I enter the school.”* Participant 07, Female 17 years old, senior 2.

Participants without disclosing their serostatus actively took part in leadership as well as in co-curricular activities like playing games. Participants feared that if their status was known publicly they would not be able to participate in school co-curricular activities normally.

*“For them knowing, that one will result* in *discrimination obviously, because if someone knows, like at this time with friends that I have been with for years, someone said, no.”* Participant 17, Male 17 years old, senior 5.

ALWH rationalized their situations as they came up with interpretations to make them stay strong or positive and adhere to ART medicines.

*“I said, I’m not going to be harassed, I’m not the one who brought that disease. I am going to be friendly* to *everyone. Even if you want me to cry, I will not.”* Participant 05, Female 19 years old, senior 5.

*“What helps me, because I see that I have lived longer because since from primary school class two I am like this and I have not yet died? So I feel happy because of that. But I keep on reminding myself that if I leave them (medicines) I can die so I take them”* Participant 13, Female 18 years old, senior 4.

### Stigma and its effects

All ALWH had either experienced stigma or feared being stigmatized due to their serostatus.

Many students had an internal stigma; they had a feeling of being gossiped about and/or rumor mongered about, being guilty, blaming themselves, or blaming their parents and having suicidal thoughts due to their status.

*“Yes, there was this particular student, this girl was born HIV positive and she reached at a time when she almost gave up her life. She was like even though I don’t have a father, I really hate my mother. I hate my mother because she infected me with HIV?”* Key informant 06, senior woman teacher

*“But you start maybe suspecting that your friend is telling some other friends that you are HIV positive. You will also start isolating yourself that maybe they know*, *yet they don’t know.”* Participant 10, Female 19 years old, senior 6.

Also, students experienced other forms of stigma such as perceived stigma and enacted stigma. Some students reported being stigmatized and discriminated against physically or verbally by either fellow students or staff.

*“ … because in the first school I used to keep my medicine along with me … there was a time where the students tried to open my case and when they opened my case, they saw medicine … they got them out … they never took them outside the dormitory but they hanged them there on my case. But then there is a friend of mine... He told me “something has happened in the dormitory because I hear students making shouting … when I went there I said “hey”... I saw my things hanged and I really was shocked … (Long pause).”* Participant 15, Male 17 years old, senior 2.

*“Yeah, they do but they don’t show it in public but they do. Like if you are going to bathe or wash your* clothes *and you use somebody’s bucket, “oh! This one used my bucket ah! So disgusting but take it”.* Participant 10, Female 19 years old, senior 6.

*“... Some teachers are very funny, they pretend to be* as *students. Also*, *they see it as something new to them though they have studied and done what … but they don’t do anything about us … You find them even talking about you instead of giving you advice … ”* Participant 18, Female 18 years old, senior 6.

Some of the ALWH experienced problems that arose as a result of being stigmatised. These problems included poor academic performance, depression, self-isolation, community isolation, loneliness, loss of hope, and poor ART adherence. Students who engaged in leadership reported discrimination if their status was known. Some students reported being de-campaigned on the grounds of being HIV positive.

*“When someone insults me when I look at the background, I look at the future, at times I see that the future is becoming very hard, so I get depressed.”* Participant 17, Male 17 years old, senior 5.

*“So of course, I could not feel okay when, while going de-campaigning me. (Yeah) No, I felt so bad. Even I called some* of *my friends; how shall we go about this thing? They said no, continue. Even there is when I wanted to say ah why should I; why should not write a letter of resignation saying that I should stop.”* Participant 01, Male 18 years, senior 4.

### Facilitators of ART adherence

There are some factors that facilitate ALWH to positively and adequately adhere to their ART that was shared by both participants and key informants. Facilitators are the factors in the boarding secondary school environment which ensures proper uptake of the medicines.

#### Willing disclosure of HIV serostatus and its gains

The majority of the students reported having willingly disclosed their serostatus to a few individuals at school who were either fellow students or members of the school staff. Students mainly disclosed their serostatus to school nurses, matrons, senior women teachers, and headteachers among school staff. Status disclosure facilitated participants to take access medications, have where to safely keep the medicine, getting time to take the medicine, and obtaining permission to go for refills.

*“So I decided to tell the matron so that when my time to take medicine reaches, she can permit me to go the dormitory to take my medicine and then come back for study.”* Participant 06, Female 16 years, senior 4.

*“Amongst the staff, it’s only the* headteacher *who knows about it. When my time for picking my drugs reaches, I go and tell him (headteacher) then he allows me to go and pick my drugs.”* Participant 08, Female 16 years old, senior 3

“Maybe *those who did not disclose but those students who disclosed to me, they normally keep their medicines at my place. I make sure that they take it (medicine) right there,”* Key informant 05, nurse*.*

Students who disclosed their serostatus also received “special consideration” from those school authorities and fellow students who knew their status which facilitated ART adherence. This special treatment included getting special meals, the time allowed to rest, exemption from some heavy physical compulsory activities for others, ability to access dormitory/ or school nurse to access his/her medications, permission to go to attend an HIV clinic for drug refills with ease, access to phones which enabled them to call their relatives in case of any difficulty, as well as timely reminders to take their medication*.*

*“For me*, *I don’t want to take medicine so I find it hard but they force me to. Because the matron cannot allow me to sleep without taking my medicine … she is the one that calls me sometimes like when I don’t want to take my medicine she* calls *and counsels me and tell me like you know you have to take your medicine.”* Participant 06, Female 16 years, senior 4.

"*My friends are helping me … They are the ones who remind me it’s time for my medicine; … time because we even don't have wall clocks at school*.” Participant 10, Female 19 years old, senior 6.

#### School mechanisms

School programs such as health talks and counselling sessions enabled students to adhere to ART medication taking. These school programs helped ALWH to understand themselves and accept their status. They coped better with life since they often planned; improvised ways of keeping their drugs safely without drawing attention and found means of taking drugs in time irrespective of whether in public places like class; thus, not often challenged by situations. Some students often resorted to leadership and co-curricular activities where they say they got confidence to live on.

*“The school normally hold health talks to students and particularly on HIV/AIDS. They normally get facilitators from nearby hospitals or even outside councilors. They come and hold health talks with the students, they go on to advise them on what should they do, how should they take themselves. Even the school has come on board to be kind to those students whenever they know such a student has such a problem, they normally become lenient at levels of to say if he needs to go home for a checkup, to pick drugs and so on really the school has come in and has adhered to the Ministry of Health guidelines’.* Key informant 03, senior woman teacher.

“ … *because I know what I’m, what I’m suffering from. I can’t stop taking my medicines because they are the ones which make me still be alive.”* Participant 05, Female 19 years old, senior 5.

#### Flexibility of ART clinics and parental/guardian support

The flexibility of ART clinics to dispense medicines lasting for a long duration helped students not to miss their pills while at school. Also, the clinic allowing parents/guardians to pick medicines for ALWH was a facilitator for adherence. Our study also noted that ALWH being able to get at ago enough pills to last a whole school term was a facilitator for adherence. This enabled the ALWH to avoid frequent travels to the clinic which could lead to unintended status disclosure and stigma which are intertwined to adherence. Parents also facilitated the students to get a special diet while at school. ALWH called back home when in hard times to get solace from parents/guardians.

*"But I usually take drugs for like 6 months such that when at school, the drugs don’t get over."* Participant 08, Female 16 years old, senior 3.

*"I don’t come back to* the *clinic, because my father is the one who takes for the whole term, so he stocks the medication to the nurse for the whole term so that I don’t get inconvenienced coming back."* Participant 17, Male 17 years old, senior 5.

*"And because I have consent with the parent, the parent sometimes sends money, I buy and cook food for her. Sometimes she says she does not like the “posho” they cook at school."* Key informant 02, health teacher.

### Barriers to ART adherence

Both ALWH and key informants reported some barriers faced in taking medicines by ALWH in boarding secondary school. These barriers affected adherence to treatment.

#### Busy boarding school schedule

The students did not have time to take their pills due to hectic school curriculum and schedule.

*“Time for taking medicine! You find for me say, I am going to be taking my medicine at ten o’clock. When* the *time of ten reaches then maybe one of the administrators comes says, “now we need you after ten before even going to the dormitories; we have a talk” which goes* directly *to twelve.”* Participant 01, Male 18 years, senior 4

*“Yes for example, the co-curriculum training* sometimes *they* interfere *with their time of taking their drugs and they find it hard to ask for permission to get out for example in the midst of training let’s say they are in pitch and time has encroached she wants to go and take the drug she could not get time so you find that girl not participating in co-curriculum because of her status”* Key informant 05, nurse.

#### Concealment of HIV status

Some ALWH preferred to conceal their HIV status to avoid being stigmatized by their peers and staff members. The students who had not disclosed their serostatus faced difficulty while getting permission to go and get drug refills from their HIV. Equally, they experienced the difficult to get permission to stay longer or access their dormitory to take their medications leading to non-adherence.

*“The other challenge is when I want to refill my medication and the deputy head-teacher who knows my* serostatus *is not there at school. So I may even spend the whole week without medicines if I don’t get a phone to communicate* with *my home.”* Participant 14 Female 16 years old, senior 3.

#### Forgetfulness

*S*tudents sometimes forgot to take their pills. This was attributed to being tired as well as there being no one to remind ALWH to take their medications. This was observed among students who had not disclosed their status to anyone at school.

*“ … Senior six students had a party and they invited us to join them, so, I went* to *the party and forgot to take my medicines.”* Participant 09, Female 17 years old, senior 3.

***“****So you find when we have exams at night and we start like around 19 hours and exams* go *up-to like 22 hours. You are from there and you are tired, sometimes you forget.”* Participant 11, Male 19 years, senior 6.

#### Stigma and lack of privacy

Stigma and lack of privacy for students to keep and take their medication was a hindrance to adherence. They tried to avoid unintended disclosure of their HIV status by taking their pills in hiding and/ sometimes missed their doses.

“*I hide myself and take the pills.* Maybe *some days, I may find very many students around my bed, and I fear to pick the pills.”* Participant 18, Female 18 years old, senior 6.

#### Limited access to drinking water and adequate food

The limited access to safe drinking water especially among ALWH who kept drugs for themselves contributed to non-adherence to treatment. Poor diet and /or an inadequate amount of food served while at school was also a challenge. Many students reported having to take their medication after food which was not appetizing enough, hence they would end up missing the drugs.

“*There are some times when you find there is no drinking water for you to take your medicines.”* Participant 08, Female 16 years old, senior 3.

*“At least maybe on what to drink because they need a lot of drinks. And you may find that for them they need fatty things, those who live with HIV positive and you may find that the school, like here we do not fry (use cooking oil) anything.”* Key informant 04, nurse

#### Medicine related issues

The packaging of medicines in plastic bottles was mentioned as a challenge as the pills would shake and make rattle noise as the students picked their daily dosages that drew attention from other students. This sometimes led to unintended disclosure. Drug side effects and pill burden were also a challenge to adherence that the students faced. These lead to non-adherence to ART treatment.

*" … and where we keep our tablets. These bottles shake and they make a lot of noise so for those who do not know that I take when they hear the bottle shake like for today … tomorrow … they will get concerned “what is that?” Some will actually want to open your case when you are not there to see what is in that thing that shakes so we usually experience that problem of bottles shaking … ",* Participant 18, Female, 18 years old, senior 6.

“*When I am reporting, I get my skirt. It has the pocket (describes inner pockets of her skirt), then I keep my drugs in the small polythene so that they cannot shake and they make* a *sound. Then I keep down there in my case then I enter the school.”* Participant 07, Female 17 years old, senior 2.

“*There is a time when she was sincere and while crying, she told me, “ I am fade up am tired of these tablets”,”* Key informant 03, senior woman teacher.

#### Limited school support mechanisms

Some schools operated as if HIV does not exist. The key informants expressed that school staff were not well equipped to handle ALWH. The students who opted not to disclose their status were often left out and there were no efforts to seek them out. There were limited awareness and sensitization about HIV to reduce stigma in schools. Some schools even did not arrange for health talks on HIV.

*“No one talks to them, no one*, *in fact, it is like they assume HIV does not exist in students. That is it. They assume it does not exist.”* Key informant 02, health teacher.

## Discussion

ALWH living in boarding schools faced challenges just like other adolescents having the disease but some challenges were unique to them. Some of ALWH participated in leadership as well as co-curricular activities. However, their engagement was attributed to majority of the students not knowing their HIV status since ALWH acknowledged that life would had been different had their serostatus been known. ALWH who contested in leadership contests were also de-campaigned on grounds of their serostatus. Like in other studies, extra co-curricular activities provided a distraction for the students [6, 7, 11].

There was willingness of ALWH to disclose serostatus to limited number of students and staff as found in previous studies [3, 6, 7, 11, 12, 16, 17]. This limited disclosure of status ensured social support from the friends and members of staff who knew their status [6, 11, 12, 16, 18]. When ALWH disclosed their HIV status, they were given special considerations like exemption from doing heavy physical work, accessing dormitories outside the allowed time to rest, getting special diet where possible, getting permission to go to attend the HIV clinic, getting special examinations in case ALWH missed one while attending HIV clinic.

Despite disclosure guaranteeing support from those who knew the student’s status; students preferred to conceal their status. This supports existing literature showing that ALWH conceal their HIV status [4, 5, 10, 19]. ALWH in boarding secondary school were depressed and also often changed schools following stigmatization. ALWH concealed their status in order not to be stigmatized, isolated or discriminated against. This often made students adapt negative ways of coping in order to prevent unintended disclosure. These were; escaping from school to get drug refills, escaping from school programs and friends to take drugs, telling lies to access their medication while at school and also to get permission for drug refills [20], disguising their identity, as well as having to hide their medication and also hide while taking their medication. We postulate that in future it might be a disaster in their lives and for the society if these ALWH develop and retain this behavioral habit of telling lies and pretending to other spheres of life.

Just like in other studies, stigma was a major challenge reported by ALWH [4, 5, 10, 21–23]. ALWH were either stigmatized by their fellow students, school staff including teachers, or by themselves. Students had either experienced stigmatization, enacted stigma, perceived stigma, or internalized stigma. Stigma or fear of stigma affected the ALWH in numerous ways including their drug adherence [6, 24]. Students reported being bullied, isolated, and discriminated against by their fellow students as a result of their status being known similar to previous studies [3, 5]. Fear of stigma also led to the concealment of HIV status which made it almost impossible for ALWH to get support from peers and school administration.

We found that a major facilitator of ART adherence among ALWH in boarding secondary schools was limited disclosure of their serostatus to colleagues and members of staff at boarding school [6, 7, 11, 16]. Disclosure to staff ensured that ALWH were able to keep their medication with staff members which guaranteed privacy for them to take their medication, got support and counselled, given permission to access their pills in dormitories outside the allowed time, got permission to go for drug refills, often got reminded to take their pills, had access to safe drinking water for taking medication and also at times would get food from these members of staff which would diversify their diet [5, 6]. However, as noted by Mutumba and others the support was mainly non-formal and a lot seemed to depend on the goodwill of particular individuals who sometimes used their own resources [6]. We found out that ALWH often were supported in form of being reminded by friends to take their medication, provided with moral support and given drinking water [5–7, 10, 18].

Although, ALWH were living in boarding secondary schools parents/ guardians played a big role in ensuring adherence to ART. As in previous studies, parental support was key in the proper adherence of ALWH to their medication [19]. Parents often facilitated the students to get a better diet. They also helped in ensuring students had their drug refills through either helping them get clearance to go back home or by bringing the drugs to school. Also parents/guardians played a pivotal role in comforting and giving social support to ALWH where necessary.

Though school mechanisms were generalized and not specifically designed to attend to ALWH in particular [3], such mechanisms like having health talks about HIV among other topics often provided solace to ALWH [6]. Self-motivation and having sufficient knowledge about their HIV condition was also a facilitator for proper drug adherence as seen in previous studies [19]. ALWH who had a purpose to live tended to plan how to adhere to ART.

ALWH in boarding secondary school experienced many barriers to their drug adherence. In line with previous studies, stigma was a major hindrance to good drug adherence for the students [6, 11, 19]. Perceived stigma meant that students needed privacy to be able to take their medication [11, 19]. However given the school setting, there was lack of privacy which is not different from the findings of previous studies [5, 11]. Students had to hide from their colleagues to take their medication and at times had to take medication from class [12, 19]. ALWH also avoided returning home for drug refills so as not to draw attention from students making their adherence poor [3].

As reported in previous studies, students’ adherence was challenged by the packaging of drugs in bottles that often drew attention to them when they were taking their drugs since they often made unnecessary rattling noise [6, 10, 25]. This was even made worse given already limited privacy in boarding schools. ALWH sometimes avoided getting their pills from these bottles or even repacked their pills so as conceal their HIV status.

Our findings showed that ALWH who had not disclosed to a member of school staff faced challenges to ART adherence arising specifically from their living in boarding school. ALWH had to miss their pills if there was no privacy in the dormitory or if the only member of staff who knew their serostatus was not around to permit them. This finding is consistent with other studies which showed that ALWH faced the challenge of having to keep their medication for themselves, lack of privacy in dormitories, having to hide from peers while taking medication having difficulties in accessing the medication from where they had kept them as well as getting permission to go for drug refills [5, 11, 12, 16, 19]. Besides, ALWH often forgot taking their medication with no one reminding them [4, 16, 26].

The busy and hectic school schedule which often occupied ALWH during times of taking their medication was another hindrance to proper drug adherence. Just as seen in other studies where participants reported a conflict in the medication and school schedule, ALWH reported skipping medication when faced with such a scenario [5, 6, 12]. In this study, we found that ALWH sometimes forgot to take their pills. This usually arose from the hectic life of boarding school leading to tiredness. This was aggravated by lack of reminders/ social support from other people. This was common in those who had concealed their serostatus. This finding is in agreement with other studies [7, 16].

Poor diet or limited availability of food in resource constrained settings like sub-Saharan Africa has been documented to be an impediment to ART adherence [3, 11, 16].]. Our findings are in agreement with this observation. ALWH sometimes missed taking their medicines on time because they had not eaten enough food or they thought that food taken at school was not nutritious enough. Another related challenge reported in our study was the scarcity of safe drinking water for taking medication. This mainly affected ALWH who were not getting “special” support from school administration. ALWH most of the time did not have money to purchase packed water or other soft drinks to use in taking their pills. The challenge of drinking water was previous reported in situations of directly observed treatment in areas with limited resources [27, 28] but not in schools. Consistent with other studies, ALWH adherence to ART was negatively affected by getting drug side effects and heavy pill burden which made students to want to stop taking medication [5, 16, 19]. ALWH were also concerned that they were taking pills without knowing if there will be prospects of them stopping.

Despite, HIV education being a part of school curriculum in Uganda [29, 30], our study found that the boarding school environment was also insensitive to the ALWH. It was reported that boarding schools operated as though there were no ALWH attending school. Health talks about HIV were not in all schools, and in schools that had them they were few. This was consistent with other studies which found out that members of staff were not sufficiently trained/equipped to handle these student’s concerns and some even breached confidentiality leading to unintended disclosure [3, 6, 7, 11]. Like in previous studies, our study affirmed that school mechanism was lacking or insufficient to help the ALWH to cope with life; the schools did not proactively look for ALWH who had not disclosed their HIV status, and initiatives aimed at helping ALWH were mainly informal and based on individual whims of the school staff [3, 6, 12, 18] .

### Study strengths and limitations

We recruited a purposive sample of participants from those attending HIV clinics in Bushenyi thus limiting the transferability of our study findings. Thus the experiences of ALWH in boarding secondary schools in Bushenyi but not getting treatment from health facilities in Bushenyi were missed. One-on-one interviews with participants had the possibility of introducing reporting bias. However, this was minimized by using interviewers who were previously unknown to participants. Self-reports were the only method of assessment yet it has been observed that adolescents are not always truthful when providing self-reports [31]. To minimize this, participants were informed about confidentiality and assured that data would be anonymized. Nevertheless, this study provides useful results that can be used with adaption in similar settings to improve the lives of ALWH.

## Conclusions

Adolescents in boarding secondary schools have unique challenges which need to be addressed. There is a need for more sensitization and awareness about HIV to reduce stigma. More in-depth qualitative research needs to be done to explore this topic further. There is need to design systems in secondary boarding school that support limited disclosure of serostatus to specific staff members as this was seen to facilitate adherence and associated with better experience by ALWH.

## Data Availability

The datasets generated and/or analysed during the current study not publicly available due to the restrictions statement in our study consent forms. However, de-identified data can be made available on reasonable request made to the corresponding author.

## Abbreviations

ALWH: Adolescents living with HIV/AIDS
HIV: Human immune virus
AIDS: Acquired immune deficiency syndrome
ART: Antiretroviral therapy
HEPI-TUITAH: Health professional education partnership initiative -transforming Ugandan institutions training against HIV/AIDS
